# Including diverse and admixed populations in genetic epidemiology research

**DOI:** 10.1002/gepi.22492

**Published:** 2022-07-16

**Authors:** Amke Caliebe, Fasil Tekola‐Ayele, Burcu F. Darst, Xuexia Wang, Yeunjoo E. Song, Jiang Gui, Ronnie A. Sebro, David J. Balding, Mohamad Saad, Marie‐Pierre Dubé, IGES ELSI Committee

**Affiliations:** ^1^ Institute of Medical Informatics and Statistics Kiel University and University Hospital Schleswig‐Holstein Kiel Germany; ^2^ Epidemiology Branch, Division of Population Health Research, Division of Intramural Research, Eunice Kennedy Shriver National Institute of Child Health and Human Development National Institutes of Health Bethesda Maryland USA; ^3^ Center for Genetic Epidemiology University of Southern California Los Angeles California USA; ^4^ Public Health Sciences Division Fred Hutchinson Cancer Research Center Seattle Washington USA; ^5^ Department of Mathematics University of North Texas Denton Texas USA; ^6^ Department of Population and Quantitative Health Sciences Case Western Reserve University Cleveland Ohio USA; ^7^ Department of Biomedical Data Science, Geisel School of Medicine, Dartmouth College One Medical Center Dr. Lebanon New Hampshire USA; ^8^ Department of Radiology Mayo Clinic Jacksonville Florida USA; ^9^ Melbourne Integrative Genomics, Schools of BioSciences and of Mathematics & Statistics University of Melbourne Melbourne Australia; ^10^ Qatar Computing Research Institute Hamad Bin Khalifa University Doha Qatar; ^11^ Neuroscience Research Center, Faculty of Medical Sciences Lebanese University Beirut Lebanon; ^12^ Department of Medicine, and Social and Preventive Medicine Université de Montréal Montréal Québec Canada; ^13^ Beaulieu‐Saucier Pharmacogenomcis Centre Montreal Heart Institute Montreal Canada

**Keywords:** admixture, diversity, genetic association, genome‐wide association study, inclusion

## Abstract

The inclusion of ancestrally diverse participants in genetic studies can lead to new discoveries and is important to ensure equitable health care benefit from research advances. Here, members of the Ethical, Legal, Social, Implications (ELSI) committee of the International Genetic Epidemiology Society (IGES) offer perspectives on methods and analysis tools for the conduct of inclusive genetic epidemiology research, with a focus on admixed and ancestrally diverse populations in support of reproducible research practices. We emphasize the importance of distinguishing socially defined population categorizations from genetic ancestry in the design, analysis, reporting, and interpretation of genetic epidemiology research findings. Finally, we discuss the current state of genomic resources used in genetic association studies, functional interpretation, and clinical and public health translation of genomic findings with respect to diverse populations.

Definition of terminologies used in the manuscript

**Additive genetic effect**: the total effect, either over alleles at a locus or over loci, equals the sum of the effects of the individual alleles or loci.

**Admixture mapping**: a gene mapping approach for phenotypes that exhibit ancestry differences used to identify associations between local ancestry and the phenotype in a sample of admixed individuals.

**Causal inference**: a process to study the causal effect of a particular factor on an outcome of interest based on data by relying on assumptions, study designs, and estimation strategies.

**Haplotype phasing**: the process of statistically inferring an individual's

genome into maternally and paternally inherited segments.

**Heritability**: a measure of the extent to which genetic variation explains phenotype differences.

**Mendelian randomization**: a causal inference approach that uses genetic variation as a natural experiment to infer causal relationship between modifiable risk factors and health outcomes.

**Meta analysis**: integrated analysis of data from different studies to answer a research question.

**Mixed models**: statistical models that include fixed effects and random effects for which the variance matrix is specified. In GWAS, the effect of a locus of interest is often modelled as fixed and is the target of inference, while the genome‐wide additive genetic contribution to the phenotype is modelled as a random effect, with the variance specified by a measure of relatedness.

**Population bottleneck**: a short period of low population size during the history of a population that leads to reduced genetic variation.

**Principal component analysis**: a dimension‐reduction technique that replaces a large number of correlated variables with uncorrelated linear combinations of the original variables. In genetic studies, the first few principal components typically reflect population structure.

**QTL mapping**: a method to identify genetic loci that are associated with a quantitative trait such as height or a measure of gene expression.

**Structural variants**: refers to a broad range of genome variations including insertion, deletion, inversion, duplication, translocation, and copy number variation.

## 1 INTRODUCTION

The field of genetic epidemiology is relatively young, but it has grown rapidly alongside the accelerating technological advances in genomics. The number of genome‐wide association studies (GWAS) published has grown 40‐fold in the past decade, with over 5000 unique publications and now nearly 300,000 associations reported in the GWAS catalog (Buniello et al., 2019). However, the body of research in genetic epidemiology is limited by an under‐representation of non‐European populations (All of Us Research Program Investigators et al., 2019; Landry et al., 2018; Popejoy & Fullerton, 2016). From a social perspective, filling this gap is essential to ensure equitable health care worldwide (Popejoy et al., 2018) and from a health research perspective, embracing ancestral diversity in genetic studies offers more opportunities to improve our understanding of the etiology of diseases (Peterson et al., 2019; Rosenberg et al., 2010). The study of population differences in disease burden and disease‐associated genetic variants can help identify functional genetic variants and any underlying evolutionary factors. One example is X‐linked dystonia parkinsonism caused by a founder mutation originating from Panay Island in the Philippines (Lee et al., 2001). Another example identified from studying admixed populations is kidney disease due to *APOL1* variants which may have risen to high frequency in parts of Africa to provide protection against African sleeping sickness (Yusuf et al., 2021). Genetic analyses of admixed populations can provide novel evolutionary insights about demographic events giving rise to phenotypic diversity (Skoglund & Mathieson, 2018) and the origin of traits (Xu et al., 2017). Linkage‐disequilibrium (LD) differences across populations are useful to refine the resolution of GWAS signals to a smaller number of potential causal variants (Helgason et al., 2007; Schaid et al., 2018; Wojcik et al., 2019) and can be leveraged to improve the accuracy of genotype imputation (Wojcik et al., 2018). Increasing ancestral diversity in genetic studies is intimately tied to advancing our knowledge of genetic and environmental factors and their potential interactions.

Addressing the existing under‐representation of non‐European populations in genetic epidemiology requires concerted efforts to conduct inclusive research, including diversifying recruitment, advancing data analysis approaches, and improving genomics resources necessary to support research based on diverse and admixed populations. Even the best analysis methods cannot outweigh a scarcity of data, and only sufficient and high‐quality data can lead to meaningful results (Buniello et al., 2019). Increasing the participation of underrepresented communities in these efforts can increase diversity and help prioritize research topics that are most relevant to individual communities, while addressing the challenges of developing frameworks for research consent, data sharing mechanisms and the return of results that respond to the communities’ needs (Hindorff et al., 2018). Lessons on these dynamics can be drawn from previous successful examples of recruiting underrepresented populations in low‐ and middle‐income settings and minorities in the United States (Horowitz et al., 2017; International HapMap Consortium, 2004; Tekola et al., 2009a). Regarding analysis methods, it is crucial that they are sufficiently flexible to enable the inclusion of diverse genomes. This is an area of active development, improving upon existing genome‐wide analytic approaches, such as mixed models, meta‐analyses, and admixture mapping Box 1 (Peterson et al., 2019). To improve our genomics and bioinformatics infrastructure, better reference populations are necessary, as the accuracy of haplotype phasing and genotype imputation heavily relies on these resources (Kowalski et al., 2019). Working toward this goal, more diverse genomics resources have recently been developed, and researchers are encouraged to consider those that appropriately fit their study population.

**Figure 1:**
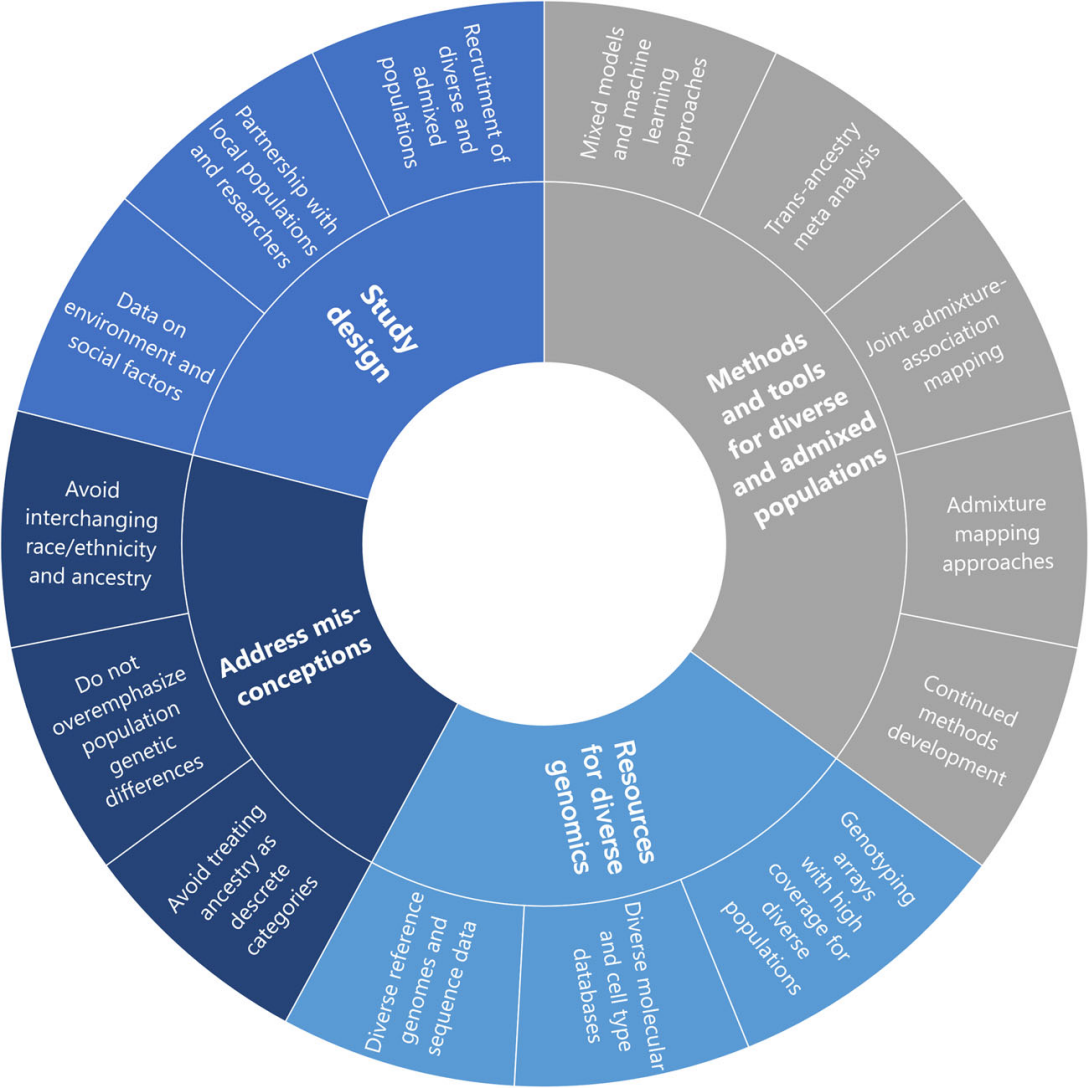
Key elements to address diversity in genetic epidemiology research

In the past year, as members of the Ethical, Legal and Social Implications (ELSI) committee of the International Genetic Epidemiology Society (IGES), we have worked together to provide guidance and support to our community for the conduct of research that is more inclusive. We released a short guideline as a website statement on the use and reporting of race/ethnicity/ancestry in IGES abstracts and papers to promote inclusion diversity and equity (IGES ELSI Committee, 2021). Further building on that effort, we here offer perspectives on methods, analysis tools, and applications that enable the conduct of inclusive genetic epidemiology research (Figure 1), including admixed and ancestrally diverse populations.

## 2 BACKGROUND AND BASICS

### 2.1 What is captured by genetic ancestry versus race/ethnicity

Here, we define *genetic ancestry* as referring to an individual's biological ancestors. Genetic ancestry can refer to ancestral groups based on the continent of residence from the majority of ancestors over many generations (e.g., African, Asian or European ancestry), or it can be more specific (e.g., West‐Central African, East Asian, or Northern European ancestry). All individuals have ancestors originating from multiple different locations at different time depth; as such, it can be more accurate to refer to proportions of genetic ancestries (e.g., 20% African ancestry) (Baker et al., 2017; Shriner et al., 2014). Information on genetic ancestry is sometimes sought from questionnaires for example by asking for country of origin of ancestors. However, self‐reported ancestry is likely to be imprecise, as study subjects may vary in the knowledge of their genetic ancestry and can choose to describe it at various levels and using differing terms (Risch et al., 2002). A variety of clustering techniques described below can be used to infer ancestry based on study participants’ genetic data, which can be more informative than self‐reported ancestry.

We will use the term *ethnicity* to refer to a social group identity that is based on shared characteristics, such as cultural traditions, ancestry, language, religion, or social experiences. Ethnicity may vary according to geographical regions and it can traverse different social classes (Peoples & Bailey, 2018). For example, French‐Canadians represent a subgroup of the Canadian society who share a common language and culture. Individuals living in the United States with measured proportions of African ancestry as low as 1% identify themselves as African American (Bryc et al., 2015; Kodaman et al., 2013). Further, some individuals from Africa are more genetically different from many Africans than from many Europeans due to the high genetic diversity within Africa (Gurdasani et al., 2015; Tishkoff et al., 2009; Yu et al., 2002). The shared social experiences of an ethnic group can have health impacts over time. As a consequence of systemic racism and segregation, Hispanic/Latino and African American individuals may be exposed to higher levels of stressors that are of economic, psychosocial, physical and environmental origin (Sternthal et al., 2011; Williams et al., 2019). To the extent possible, specific social determinants should be directly measured and accounted for in scientific enquiries, while ethnicity can be used to loosely capture other unaccounted social factors impacting health. For example, atopic dermatitis is more common among African American children, however, no ancestry‐related genetic effects could explain the increased disease prevalence in this population, suggesting that underlying social factors captured by self‐reported ethnicity may be responsible for the difference (Dikilitas et al., 2020).

The term *race* has often been used to refer to biological differences between human groups, allegedly underpinned by genetic differences (Borrell et al., 2021; Yudell et al., 2016). But it is now understood that human variation is complex, with no generally accepted classification system, and that race is largely a social construct similar to ethnicity that may or may not be correlated with biological traits. Care must be taken when using the term race in genetic epidemiology, as it can lead to an overemphasis on biological differences between groups of individuals. In particular we should avoid using controversial terms such as “Caucasian,” originating from the 18th century in support of racist arguments (Birney et al., 2021; Race, Ethnicity, and Genetics Working Group, 2005). Genetic epidemiology investigations can be easily misinterpreted or co‐opted to promote messages of biological determinism or inferiority, and it is our responsibility as scientists to ensure that we communicate findings in a way that avoids such misinterpretations. Genetic differences between groups may be of interest in population genetics research or in genetic epidemiology research of diverse and admixed populations. For many research questions, however, the reasons for considering ethnicity, race or ancestry are to adjust for non‐genetic effects and ensure that biological interpretations and public health interventions are applicable across broad populations (Birney et al., 2021). The reasons and intent for the inclusion of ethnicity, race or ancestry must be stated clearly in scientific reports. It is also important to recognize that race/ethnicity may not adequately capture lived experiences of individuals that vary within a population group. Efforts should be strengthened to standardize psychological, demographic, and socioeconomic information, which can be integrated with genomics to ensure the conduct of more accurate and informative research. As we continue to progress toward the conduct of more inclusive and diverse studies in genetic epidemiology, we should strive to conduct health research based on the direct measurements of health factors that will take the science beyond notions of race (Borrell et al., 2021; Yudell et al., 2016).

### 2.2 Forces that shape worldwide genetic diversity in human populations

The genetic diversity that exists worldwide today results from the interplay of population genetic forces, mainly mutation, recombination, genetic drift, selection, and migration. Underlying all genetic variability are *mutations* that create new alleles in individuals. Single‐site substitutions occur at a rate of about 10^‐8^ per base pair and per generation, creating single nucleotide variants (SNVs), which are commonly used in genetic epidemiology research. However, this mutation rate is highly variable and can depend on genetic and epigenetic context, sex, and age of the father at the time of conception, among other factors (Segurel et al., 2014).

*Recombination* occurs during meiosis, when crossing over between homologous chromosomes generates new chromosomal combinations that are passed on to the next generation, breaking down the LD between loci. The recombination rate varies across the genome and there appears to be subtle differences in recombination rates between populations (Graffelman et al., 2007; Penalba & Wolf, 2020; Wegmann et al., 2011).

*Genetic drift* is the variation in allele frequencies due to stochastic sampling of alleles across generations. This is relevant for genetic epidemiology studies in populations that are currently small and isolated, or larger populations that have grown rapidly from a small size, such as the out‐of‐Africa bottleneck experienced by the ancestors of non‐African populations. Whereas genetic drift usually shifts allele distributions over a relatively long period of time, *selection* can do so more rapidly. Positive and negative selection can quickly cause fixation or removal of alleles, whereas balancing selection maintains the polymorphism. Selection can act differently in different populations due to varying environments, resulting in diverse allele frequencies between populations. Because selection typically acts through differential reproductive success, it has less of an impact on diseases with later age at onset, such as Parkinson's disease, or Alzheimer's disease.

Well‐known examples of traits where selection played a major role include skin color, lactase persistence and sickle cell trait. It should be noted that such traits are not good indicators of the genetic ancestry of an individual. For example, an individual may fall into different categories of skin color in different geographic regions (Marcheco‐Teruel et al., 2014; Parra et al., 2003). In Africa, skin color is diverse and genetic signatures of selection for both lighter and darker skin color have been reported (Crawford et al., 2017; Genomes Project Consortium et al., 2015). Similarly, lactase persistence has evolved via different genetic mechanisms and in different parts of the world where cattle‐herding and milk consumption are culturally embedded (Heyer et al., 2011; Segurel & Bon, 2017; Tishkoff et al., 2007; Wiley, 2020).

Another factor that influences worldwide genetic diversity is *migration*. Recent large‐scale migration leads to population mixing that is referred to as admixture. These scenarios can have different consequences for genetic studies and must be treated appropriately during data quality control analyses and interpretation. For example, recent migration can lead to long‐range LD as recombination has not had time to do its mixing work, and recently admixed populations can present an excess of heterozygotes. While drift and selection can cause allele frequencies to diverge across populations, sharp genetic boundaries between neighboring populations are rare. Instead, migration causes allele frequencies to change smoothly with distance, which is referred to as a *cline*. This is exemplified by European populations where migration is known to have played a major role, and many allele frequencies vary smoothly across the continent, even across language boundaries and national borders (Lao et al., 2008).

## 3 METHODS AND TOOLS FOR INCLUDING DIVERSE AND ADMIXED POPULATIONS

A variety of methods and tools are available for analyzing GWAS (M. H. Wang et al., 2019). The simplest ones are performed on a single‐variant basis and use linear or logistic regression such as provided by PLINK (Chang et al., 2015). Linear or logistic mixed models are able to incorporate correlation structure between individuals arising, e.g., by relatedness or population structure (H. Chen et al., 2016; Kang et al., 2010; Zhou & Stephens, 2012). Recently, Bayesian approaches have become more common (Kaplan et al., 2020; Lock & Dunson, 2017). Utilizing the results of GWAS, polygenic scores (PGS) summarize the effects of multiple genetic variants in one variable. They are usually generated by selecting a number of most influential variants and adjusting for LD. For the calculation of individual PGS, the genotype dosage of variants is weighted by the variant effect sizes, which are either directly taken from GWAS or recalculated using refined methods (Hindorff et al., 2018).

Post GWAS investigations aim to detect the functional effects of genetic variants. Here, analyses of expression quantitative trait loci (eQTL) and of tissue‐ or cell‐specific expression are frequently performed. Other approaches include colocalization, which aims to discover genes for which a single causal genetic variant underlies two phenotypes (Giambartolomei et al., 2014; Hormozdiari et al., 2016) and prediction of pathogenicity (Knecht & Krawczak, 2014). Populations with diverse and admixed ancestry due to recent migration require specific analytic considerations. Whereas causal mechanisms are assumed to be the same across populations, the between‐population differences in LD between a causal variant and marker can lead to large differences in observed effect sizes. Because the LD structure can be considerably different between populations, methodological problems arise when jointly analyzing GWAS data from cohorts of different ancestry or of admixed populations. Additional complications can arise due to gene‐environment interaction. Here, we highlight some of the methodological challenges in genetic epidemiology studies and present analytic strategies that may be used to mitigate these challenges. We also point out limitations, highlighting areas that require further methodological development.

### 3.1 Population structure

Systematic allele frequency differences in subgroups of individuals within a study population, known as population structure, can confound genetic association studies, leading to false positive findings (Patterson et al., 2006; Price et al., 2006). Principal component analysis (PCA) is commonly used in GWAS to adjust for population structure with or without the addition of external reference samples. Plots of the first few principal components (PCs) can be used to identify subpopulations, and including PCs as covariates in regression models can adjust for confounding due to low levels of population structure (Lazaridis et al., 2014). While PCA is good at capturing global ancestry information across autosomal chromosomes, it is less effective at capturing different forms of population structure such as admixture which can inform association analysis. Another drawback is that determining the number of PCs to include as covariates is ad hoc and using too few or too many PCs can lead to spurious associations and loss of power (J. Zhang, 2010). Whereas autosomal variants are inherited from both mother and father, the Y chromosome is passed from father to son only and the mitochondrial DNA is passed from a mother to all her children. Depending on how admixture, intermixing and the asymmetry of intermixing occurred in previous generations, an individual's Y‐chromosomal and mitochondrial ancestries may differ substantially from ancestries at autosomal loci.

Population structure can influence trait association, causal inference, heritability and trait prediction models. Statistical methods must be adapted to both the scientific question and the underlying population structure. For example, if ancestry is associated with an environmental factor that is causally associated with the trait, adjustment using PCs alone may insufficiently account for confounding. Ideally, the environmental factor should be included in the model, but this information is often unavailable (Lawson et al., 2020). Of note, the detection of population structure depends on sample size. Only broad population structure can be detected in small samples, whereas more fine‐scale structure can be evident in larger studies.

Large genetic drift that can occur in small populations or strong bottlenecks can lead to allele frequency differences for which adjusting for PCs may not be effective in accounting for population structure (Lawson et al., 2018, 2020). Selection may also impact allele frequencies differently between populations, which could affect genetic associations. Especially susceptible to population structure are studies of rare genetic variants, where frequencies are highly variable across populations, and studies of haplotypes that are highly polymorphic. LD structure varies widely between populations as well. As such, investigations of rare variants or those dependent on LD, such as GWAS and PGS, can result in population‐dependent results. In addition, ancestry‐related assortative mating in a population can contribute to maintaining population stratification and can lead to long‐range LD, which may further impact genetic association studies (Risch et al., 2009; Sebro et al., 2010).

### 3.2 Ancestry estimation and analysis approaches for admixed populations

Demographic events such as migration and mating between isolated populations with distinct genetic structures lead to admixed populations with mosaic chromosomal segments from each isolated population (Shriner, 2017). Admixture is a common feature of human history and has shaped present‐day human genetic variation (Hellenthal et al., 2014; Patterson et al., 2012; Shriner et al., 2016). One of the earliest known examples of archaic admixture in hominins is that of *Homo sapiens* and *Homo neanderthalensis (*Durvasula & Sankararaman, 2020; Sankararaman et al., 2014; Wall et al., 2013). Recent admixture triggered by forced and voluntary human migration and mixing has introduced major gene flow among geographically isolated populations (Micheletti et al., 2020). Examples of recently admixed populations include African Americans, an admixed population of African and European ancestry (Micheletti et al., 2020), populations in Central and South America, which are admixed populations of Amerindigenous, European and African ancestry (Bryc et al., 2010; Kehdy et al., 2015), and multi‐ancestral admixed populations in South Africa (Kodaman et al., 2013). Koehl and Long investigated 17 admixed populations in the Americas and showed that their genetic diversity reflected varying contributions of admixture and genetic drift (Koehl & Long, 2018). In genetic epidemiology studies, admixed populations present unique challenges for association analyses between genotypes and phenotypes when the causal genetic variant has different frequencies across the ancestral populations. Individuals belonging to an admixed population group vary in their distributions of ancestry proportions. This genetic heterogeneity and complex substructure can confound associations and lead to spurious findings unless properly accounted for in the analyses (Landry et al., 2018; Thornton & Bermejo, 2014).

Several statistical tools have been developed for ancestry inference and admixture analysis. Statistical tests such as the D test (Durand et al., 2011) and *f* test (Patterson et al., 2012) are useful to detect the existence of admixture and the direction and magnitude of gene flow between parental and admixed populations. An estimate of the genomic contributions of different ancestral populations in an admixed population can be inferred at global or local levels. The *local ancestry* is the ancestry at a specific genomic region, while the *global* ancestry of an individual is the genome‐wide distribution of its *local* ancestry (Mersha, 2015). In Table 1, we summarize important characteristics of some of the many approaches that have been proposed to estimate ancestry proportions. Comparison of admixture mapping tools and detailed reviews have previously been published (Padhukasahasram, 2014; Seldin et al., 2011). Genetic ancestry inference techniques have different assumptions and limitations, and a careful selection of the appropriate method for a given study setting is necessary. The robustness of results can be assessed by comparing multiple methods. In general, differences between admixture inference tools can be due to the underlying model assumptions such as whether LD between SNPs is considered or not, applicability to multi‐way admixed populations, accuracy, computational speed, the need for dense genotypes and whether the input genotype data need to be phased.

**Table 1:** Software and methods for deriving population structure

Software	Description	Software Platform	Input	Notes	References
*Local ancestry software* a					
HAPMIX	HMM Two ancestral populations	C++	Reference haplotypes Unphased genotypes	Haplotypes of each admixed individual are viewed as being sampled from the reference populations.	Price et al. (2009)
SEQMIX	HMM Two ancestral populations	C++	Allele frequencies for reference populations Genetic distances VCF for gene sequence variations	Using aligned bases of combined off‐target and on‐target sequence reads directly to infer whole‐genome ancestry in admixed samples	Hu et al. (2013)
LAMP‐LD	HMM Multiple (≥2) ancestral populations	C++	Reference haplotypes Unphased genotypes	Using nonoverlapping windows No transitions between ancestries within each window An order of magnitude faster than HAPMIX	Baran et al. (2012)
RFMix	Linear‐chain conditional random field (CRF) model + random forests Multiple (≥2) ancestral populations	C++	Reference haplotypes VCF for gene sequence variations	Dividing each chromosome into windows and inferring local ancestry within each window by using a CRF parameterized by random forests trained on reference panels	Maples et al. (2013)
MULTI‐MIX	HMM Multiple (≥2) ancestral populations	C++	Either phased or unphased data	A multivariate normal model on haplotype probabilities given ancestry and an HMM on how ancestry changes along a chromosome	Churchhouse and Marchini (2013)
ALDsuite	HMM + PCA Multiple (≥2) ancestral populations	R	Phased haplotypes	Using an HMM to model switches between ancestral states across each individual's\ chromosomes; using PCs to approximate admixture LD; offering both local and global ancestry inference using dense marker data	Johnson et al. (2015)
*Global ancestry software*					
fineSTRUCTURE	HMM, MCMC Multiple (≥2) ancestral populations	C++/R	Phased haplotypes Recombination rates	A haplotype segment viewed as the ‘recipient’ of genetic material from nearest neighbor ‘donor’ haplotype segments; such donor‐recipient relationships created for every haplotype in the data	Lawson et al. (2012)
ADMIXTURE	Bayesian clustering approach Multiple (≥2) ancestral populations	C++	Unphased genotypes	Estimating individual ancestries by computing maximum likelihood estimates in a parametric model	Alexander and Lange (2011)
IPCAPS	PCA Multiple (≥2) ancestral populations	R	Unphased genotypes	Building on the iterative pruning PCA framework that systematically assigns individuals to genetically similar subgroups. It can be used with transcriptome or epigenome data.	Chaichoompu et al. (2019)

Abbreviations: HMM, hidden Markov model; MCMC, Markov chain Monte Carlo; PCA, principal component analysis; VCF, variant call format.

^a^ Global ancestry proportion can be calculated as the genome‐wide average of local ancestry proportions.

The underlying models of fineSTRUCTURE (Lawson et al., 2012), ADMIXTURE (Alexander & Lange, 2011), and IPCAPS (Chaichoompu et al., 2019) estimate global ancestral proportions, while HAPMIX (Price et al., 2009), SEQMIX (Hu et al., 2013), LAMP‐LD (Baran et al., 2012), RFMix (Maples et al., 2013), MULTI‐MIX (Churchhouse & Marchini, 2013), and ALDsuite (Johnson et al., 2015) provide inference on local ancestry. To infer ancestry, SEQMIX (Hu et al., 2013) uses sequence reads from exome or targeted sequencing of selected regions of interest that have not mapped to the intended regions. HAPMIX models background LD and describes how the haplotypes of admixed individuals relate to those in the ancestral populations. Unlike HAPMIX and SEQMIX that are only applicable to admixtures of two ancestral populations, the other listed software packages in Table 1 are useful for estimating ancestries of populations that are admixtures of two or more ancestral populations. MULTI‐MIX estimates local ancestry using a model on background LD and can handle either phased or unphased genotype data. A limitation of many current methods is that they require large LD reference panels, which remain under‐representative of individuals from minority population groups. RFMix can utilize ancestry information contained within admixed samples themselves rather than an LD reference panel and is also fast enough to analyze tens of millions of SNPs (Maples et al., 2013; Uren et al., 2020).

In the analysis of genetic data from admixed populations, accounting for local ancestry in addition to the traditional approach of accounting for global ancestry improves the accuracy of result (Landry et al., 2018; Liu et al., 2013; Zhang & Stram, 2014). However, this comes at the cost of longer computation time. Therefore, a practical recommendation is to consider a two‐stage approach in which the first model includes adjustment for global ancestry and the second model includes local ancestry adjustment on candidate loci (Gay et al., 2020). On the other hand, when causal genetic variants differ between ancestral populations, admixture mapping methods can leverage admixed populations to identify ancestry‐specific loci associated with phenotypes (Patterson et al., 2004). Methods that jointly test genotype‐phenotype associations and admixture mapping achieve higher power in genotype‐phenotype tests and reduce the testing burden in ancestry‐phenotype tests (W. Chen et al., 2015; Pasaniuc et al., 2011; Shriner et al., 2011).

### 3.3 Multi‐ancestry and meta‐analysis GWAS approaches

Meta‐analysis of individual studies grouping individuals by ancestral populations can be an effective way to circumvent issues related to population stratification and has similar power as analyses pooling individual‐level data (Lin & Zeng, 2010; Willer et al., 2010). Traditional meta‐analyses assume ancestral homogeneity between populations, which may not be appropriate for multi‐ancestry studies where heterogeneity is common. Meta‐analysis methods such as MR‐MEGA, MANTRA and GWAMA aim to address this issue by modeling allele effect heterogeneity between populations (Table 2). However, stratification by ancestral populations can be problematic, as genetic ancestry is a continuum and individuals do not distinctly group into a single ancestral population, with the majority of individuals having mixed ancestry. Because of this, the practice of grouping individuals into single homogeneous populations, which typically entails excluding those who do not group into a distinct population may not be optimal. An alternative is to conduct a joint analysis across ancestries while allowing for differences in trait variance across groupings based on population characteristics as a random variable in a mixed model (Wojcik et al., 2019).

**Table 2:** GWAS methods for inclusion of multi‐ancestry populations

Method	Description	Outcome (binary/quantitative)	Software platform	Suitable for *N* > 100 k scale analyses	Notes	Reference
*Pooled approaches accounting for relatedness and ancestry*						
REGENIE	Ridge regression Generalized linear model	Binary Quantitative	C++	Yes	Reduces computational burden of calculating GRM by instead accounting for local LD within blocks with “local polygenic scores” calculated using ridge regression Supports Firth and SPA for binary traits Can analyze multiple phenotypes in parallel	Mbatchou et al. (2021)
fastGWA	Linear mixed model^a^,^b^	Binary Quantitative	C++	Yes	Uses grid‐search‐based REML algorithm to estimate a sparse GRM Implemented in GCTA	Jiang et al. (2019)
BOLT‐LMM	Linear mixed model^a^,^b^	Binary Quantitative	C++	Yes	Models non‐infinitesimal genetic architecture via a Bayesian mixture prior on variant effect sizes	Loh et al. (2015) Loh et al. (2018)
SAIGE	Logistic mixed model^a^	Binary Quantitative	R	Yes	Controls for unbalanced case‐control ratios using SPA	Zhou et al. (2018)
SUGEN	Generalized estimating equations	Binary Quantitative	C++	Yes (?)	Accounts for unequal inclusion probabilities using weighted version of GEEs	Lin et al. (2014)
GENESIS	Logistic mixed model^a^ Linear mixed model^a^	Binary Quantitative	R	Yes (?)	Allows for multiple variance components Part of a suite of tools utilizing GDS format	Gogarten et al. (2019)
GMMAT	Logistic mixed model^a^	Binary Quantitative	R	No	Utilizes GDS format Beta coefficients not provided for GWAS	H. Chen et al. (2016)
GEMMA	Linear mixed model^a^	Quantitative	C++	No	Exact method	Zhou and Stephens (2012)
EMMAX	Linear mixed model^a^	Quantitative	C++	No	Approximation method	Kang et al. (2010)
Tractor	Local ancestry aware logistic regression model	Binary	Python, Hail	Yes	Facilitates the inclusion of admixed individuals by leveraging local ancestry	Atkinson et al. (2021)
*Meta‐analysis approaches enabling multiple ancestries*						
MANTRA	Bayesian partition model	Binary Quantitative	Fortran (?)	Yes	Models allelic heterogeneity between populations using a hybrid fixed and random effects approach	Morris (2011)
MR‐MEGA	Multi‐dimensional scaling	Binary Quantitative	C++	Yes	Models heterogeneity in allelic effects as a function of pairwise mean allele frequency differences between populations	Magi et al. (2017,2020)
TransMeta	Kernel‐based random‐effects model	Binary Quantitative	R	Yes	Models heterogeneity based on the correlation structure of allelic effects, which are treated as random variables Beta coefficients not provided	Shi and Lee (2016)
GWAMA	Random‐ or fixed‐effects meta‐analysis	Binary Quantitative	C++	Yes	Models allelic heterogeneity using random‐effects in the presence of heterogeneity and fixed effects otherwise	Magi and Morris (2010)
METAL	Fixed effects meta‐analysis	Binary Quantitative	C++	Yes	Meta‐analyzes either 1) P‐values and effect directions or 2) effect size estimates weighted by standard errors.	Willer et al.(2010)
METASOFT	Random‐ or fixed‐effects meta‐analysis	Binary Quantitative	Java	Yes	Includes conventional models and a random effects model that increases power under heterogeneity.	Han and Eskin (2011)

Abbreviations: GDS, genomic data storage; GEE, generalized estimating equations; GRM, genetic relationship matrix; GWAS, genome‐wide association studies; LD, linkage disequilibrium; REML, restricted maximum likelihood; SPA, saddlepoint approximation.

^a^ Mixed models require either an externally pre‐calculated or internally calculated GRM.

^b^ Note that linear mixed models are not designed to analyze binary traits and can have inflated type I error rates.

### 3.4 Mixed models and relatedness between individuals

Unaccounted relatedness can lead to spurious association signals due to false assumptions of independence between individuals (William & David, 2009). To address relatedness in association analysis, there are analysis approaches that can pool together individuals of diverse ancestry by using mixed models to incorporate a measure of genetic similarity, such as the popular genetic relationship matrix (GRM), as a random effects variance‐covariance matrix (Table 2). Genetic relatedness is a fundamental concept in human genetics, and approaches for defining and estimating relatedness have been extensively described previously (Speed & Balding, 2015; Thompson, 2013; J. Wang, 2016). Speed and Balding (2015) prefer the term *genetic similarity matrices* from the perspective that genetic similarity is what is captured from such approaches rather than relatedness itself. Ramstetter et al. (2017) evaluated 12 pairwise relatedness inference methods using genome‐wide data, including for example KING (Manichaikul et al., 2010) and PC‐Relate (Conomos et al., 2016), which provide an excellent reference. In Table 3, we highlight some additional GRM computation tools recently developed, focusing on those that accommodate samples of heterogeneous ancestry and sequence data, excluding IBD segment inference tools. Many pooled GWAS analysis tools using mixed models include a GRM computation feature. Pooled GWAS analysis methods that also include the estimation of a GRM include BOLT‐LMM (Loh et al., 2015, 2018), SAIGE (Mogil et al., 2018), SUGEN (Lin et al., 2014), GENESIS (Naseri et al., 2019), and GEMMA (Zhou & Stephens, 2012, 2012) (Table 2).

**Table 3:** Tools for genetic relatedness matrix (GRM) estimation for inclusion of multi‐ancestry populations.

Tool	Method type	Description	Software language	Input file type and external info needed	Output	Notes	Reference
*For called genotype (SNP) data only*							
SNPRelate	Allele frequency‐based IBD estimate	For multi‐core symmetric multiprocessing computer architectures For principal component analysis (PCA) and relatedness analysis	R with C/C++ kernel	GDS/VCF/NetCDF	IBD 0,1,2 GRM matrix	Can be used for population structure with KING‐robust method	Zheng et al. (2012)
RaPID	IBD‐segment based IBD estimate	Based on the positional Burrows‐Wheeler transform Linear time search for shared segments in an arbitrarily large cohort	C++	VCF Genetic map	IBD 1,2	Assumes randomly distributed genotyping errors and correct genetic map	Naseri et al. (2019)
PedKin	Pedigree‐based	Set of algorithms for computing the kinship coefficient of a set of individuals with a known pedigree	C++	PLINK ped Inbreeding values for founders	GRM matrix	Considers the possibility that the founders of the known pedigree may themselves be inbred	Kirkpatrick et al. (2019)
IBIS	IBD‐segment based IBD estimate	Locates long regions of allele sharing between unphased individuals, detects IBD segments and infers degrees of relatedness	C++	PLINK binary Genetic map	Kinship, IBD 2	For both admixed and unadmixed samples Assumes universal allele sharing for missing data sites	Seidman et al. (2020)
popkin	Allele frequency‐based IBD estimate	Method of moments estimation for kinship and FST Practically unbiased for any population structure	R/Rcpp	Genotype matrix or PLINK BED Vector of baseline kinship values	GRM matrix	Accounts for heterogeneous samples with population structure and admixture	Ochoa and Storey (2021)
*For called genotype (SNP) data/genotype likelihood (dosage) data/sequence data*							
MAPGD	Maximum Likelihood‐based IBD estimate	Estimates of inbred‐relatedness coefficients from population genomic data	C++	BAM mfileup file from samtools	7 genotypic correlation coefficients		Ackerman et al. (2017)
SEEKIN	Allele frequency‐based IBD estimate	Estimates kinship for both homogeneous samples and heterogeneous samples using sparse sequence reads	C++	VCF Ancestry coordinates generated by LASER	GRM matrix	Accounts for heterogeneous samples with population structure and admixture	Dou et al. (2017)
TRUFFLE	IBD‐segment based IBD estimate	Integrates computational techniques and statistical principles for the identification and visualization of IBD segments	C++	VCF	IBD 0,1,2	Uses un‐phased data by skipping the haplotype phasing step and, instead, relying on a simpler region‐based approach	Dimitromanolakis et al. (2019)
NgsRelate v2	Allele frequency‐based IBD estimate	Estimates the 9 condensed Jacquard coefficients along with various other relatedness statistics from high‐throughput sequencing data	C++	BAM/CRAM, VCF/BCF, PLINK BED	IBD 0,1,2	Accounts for arbitrary inbreeding patterns within homogeneous population	Hanghoj et al. (2019)
NGSremix	Maximum Likelihood‐based IBD estimate	Maximum likelihood estimation tool of relatedness between pairs of admixed individuals from low‐depth NGS data	C/C++	PLINK binary or BEAGLE format for genotype likelihood Individual ancestry proportions and population specific allele frequencies	IBD 0,1,2	Accounts for heterogeneous samples with population structure and admixture Takes the uncertainty of the genotypes into account via genotype likelihoods and handles admixture by estimating paired ancestry proportions and including these in the model	Nohr et al. (2021)

Abbreviations: BAM, binary alignment map; BCF, BIM collaboration format; GRM, genetic relationship matrix; IBD, identity by descent (IBD 0, 1, 2 denotes the probability that two individuals have 0, 1, or 2 alleles at a locus identical by descent); VCF, variant call format.

Association analyses based on mixed models have the advantage of enabling the inclusion of individuals with recent and distant relatedness and circumvent the need to arbitrarily keep unrelated individuals only. Because pooled analyses use individual‐level data, covariates need to be consistently included across studies, which also allows the investigation of potential interactions. In such studies, it is common to additionally adjust for PCs capturing genetic ancestry, and adjustment for self‐reported race/ethnicity has been applied in previous studies to account for potential social or cultural confounders (Wojcik et al., 2019). Studies have shown that pooled approaches using mixed models control for genomic inflation (estimated using the lambda factor; an indicator of the impact of population structure and other confounders on association results [Devlin & Roeder, 1999]) better than either adjusting for PCs or excluding related individuals in generalized linear models (Y. Zhang & Pan, 2015). However, the need for individual‐level data in mixed models has the limitation of being more computationally intensive and more subject to data sharing and privacy issues than studies that rely on the meta‐analysis of GWAS summary statistics alone (Pasaniuc & Price, 2017). The use of mixed models for multi‐ancestry GWAS do leave some open questions with respect to how to best model effect size differences that can occur between populations due to the different LD structures and differences in social and environmental factors (Peterson et al., 2019). More research from a methodological perspective, either theoretical or by simulations, is warranted. More recently, a statistical method and software named *Tractor* (Atkinson et al., 2021) facilitates the inclusion of diverse and minority populations in genomic research (Table 2) by accounting for local ancestry and population differences in minor allele frequencies at each tested variant. Peterson et al., provide a useful review of quality control considerations and analysis strategies in diverse populations (Peterson et al., 2019).

### 3.5 PGS in the context of diverse populations

PGS, including polygenic risk scores (for case‐control traits), aggregate many genetic variants investigated in GWAS (Hindorff et al., 2018; Janssens, 2019). PGS have the advantage of summarizing the effects of multiple genetic variants, often with small effects, into a single variable under the assumption of additive genetic effects. PGS have been applied in biomedical and social science research (Knowles & Ashley, 2018; Pasaniuc & Price, 2017) and by direct‐to‐consumer genetic testing companies. It is expected that PGS will improve health outcomes by optimizing diagnostic screening practices and patient‐tailored treatments (Duncan et al., 2019; Torkamani et al., 2018), potentially bridging the gap between discovery of susceptibility genetic variants and clinical implementation (Wand et al., 2021), particularly when combined with conventional risk predictors (Elliott et al., 2020).

Interest in developing and applying PGS to predict genetic liability to complex traits has been fueled by new methodologies (e.g., clumping and *p* value thresholding [Choi & O'Reilly, 2019; Wray et al., 2014] penalized regression (lassosum [Mak et al., 2017]) and accounting for LD between predictors (LDpred [Vilhjalmsson et al., 2015]) and the publication of thousands of GWAS with large sample sizes (Hindorff et al., 2018; Khera et al., 2018). However, because PGS are largely derived from studies of European ancestry participants, they often have limited performance in non‐European ancestry populations (Dikilitas et al., 2020; Duncan et al., 2019; Martin et al., 2019; Popejoy & Fullerton, 2016) with few studies or traits providing evidence of trans‐ancestry portability (Conti et al., 2021; M. Wang et al., 2020). Population differences in allele frequencies of susceptibility variants, their effect sizes and LD patterns contribute to the non‐generalizability of PGS across populations (Martin et al., 2017).

Recently, approaches have been proposed to develop PGS for diverse populations. Simulations and real data applications have shown that including in PGS variants discovered in diverse ancestral populations led to reduced bias and higher genetic risk accuracy in admixed individuals (Cavazos & Witte, 2021). Another approach, XP‐BLUP, proposes a multiple‐component linear mixed model to incorporate ancestry‐specific weights to address the need for efficient prediction in minority populations (Coram et al., 2017). An approach called partial PGS, a method that estimates local genetic ancestry and applies a combination of ancestry‐specific PGS, found that when GWAS data are available for more than one ancestry, the combination of multiple partial PGS improves trait predictability in admixed individuals (Marnetto et al., 2020). The formation of PGS consortia such as The Polygenic Risk Scores Diversity Consortium (https://www.genome.gov/Funded-Programs-Projects/Polygenic-Risk-Score-Diversity-Consortium) will likely lead to further improvements in PGS performance across populations.

## 4 RESOURCES FOR INCLUDING DIVERSE AND ADMIXED POPULATIONS

Genetic resources from diverse populations, such as sequencing and genome‐wide genotyping array datasets, are important in population genetics and genetic epidemiology research, particularly as reference panels for estimating ancestry or LD. The LD structure is fundamental for many analyses in genetic epidemiology and required for, e.g., accurate imputation, colocalization and fine‐mapping. Although a diverse reference panel might be preferred for better imputation across various ancestry groups, colocalization and fine‐mapping can be performed meaningfully only if the LD structure can be inferred based on samples from an ancestrally similar population. However, existing resources have small sample sizes for non‐European ancestry populations and do not reflect global diversity. To reduce health disparities in genomic medicine, it is necessary to continue to enrich those resources with samples from diverse population groups. Key to this is to increase sample sizes for underrepresented populations by building research consortia. Highly populated regions of the world consist of diverse populations that remain underrepresented in genomics (Martin et al., 2019; Wojcik et al., 2019). Previous and ongoing global projects that have built sequence resources on diverse genomes include the 1000 Genomes Project (Byrska‐Bishop et al., 2021; Genomes Project Consortium et al., 2015), the Human Genome Diversity Project (Bergstrom et al., 2020), and the Human Genome Structural Variation Consortium (Ebert et al., 2021). Regional initiatives such as the African Genome Variation Project (Gurdasani et al., 2015), the Human Heredity and Health in Africa (H3Africa) (H3Africa Consortium et al., 2014), the Non‐Communicable Diseases Genetic Heritage Study (NCD‐GHS) consortium in Nigeria (Fatumo, Yakubu, et al., 2022), the Qatar Genome Program (Thareja et al., 2021), Japan Biobank (Sakaue et al., 2021), and the GenomeAsia100K Consortium (Wall et al., 2019) have made important contributions to genomics research for African and Asian populations. In the United States, recent large‐scale programs to accelerate the representation of minority and underrepresented populations in genomics and beyond include the NHLBI TOPMed Program (Taliun et al., 2021) and the All of US research program (All of Us Research Program Investigators et al., 2019). Admixed populations pose additional analysis challenges because neither of the population‐specific reference databases can be applied directly. For such purposes, more diverse reference panels are needed. The composition of these is non‐trivial and further research has to be launched to investigate what constitutes a meaningful reference database and under which analysis scenario for admixed populations.

A key resource for the identification and functional interpretation of disease‐associated variants in whole genome or exome sequencing analysis is the human reference genome (Lappalainen et al., 2019; Wong et al., 2020). However, the most widely used haploid reference genomes (release GRCh37/GRCh38) do not yet adequately represent the genomic diversity of human populations. This means that population‐specific rare variants, haplotypes and structural variants cannot be captured well for certain populations. This can have implications for the development of individualized therapies based on those markers (Chrisman et al., 2022). The recent Human Pangenome Reference Consortium (HPRC) is working to establish a human genome reference that reflects the existing worldwide human diversity (https://humanpangenome.org/). Future work is needed to investigate optimal usage of ancestry and admixture in genome references.

Genome function databases from diverse populations are valuable post‐GWAS tools to support the interpretation of genetic associations. Integrating GWAS loci with molecular traits, such as gene expression and methylation in trait‐relevant tissues, enables the prioritization of potentially causal genes, which can be targeted for developing molecular diagnostics and therapeutics. One approach in functional genomics uses genotype data to predict gene expression by identifying eQTL (Barbeira et al., 2018; Gamazon et al., 2015; Shriner et al., 2016), and associations between the predicted gene expression and a given trait of interest are evaluated using Mendelian randomization for causal inferences (Hauberg et al., 2017; Pavlides et al., 2016; Zhu et al., 2016). Another approach is colocalization to identify variants underpinning multiple molecular traits, such as gene expression and methylation (Giambartolomei et al., 2014; Hormozdiari et al., 2016). However, the most widely used database for integrated functional analyses of tissue‐specific gene expression targets, the Genotype Tissue Expression Portal (GTEx), consists of more than 80% European‐ancestry individuals (GTEx Consortium, 2020). The accuracy and power of gene expression prediction models improve when models are built in individuals whose ancestry is representative of the phenotyped individuals (Keys et al., 2020; Mikhaylova & Thornton, 2019; Mogil et al., 2018). Diverse samples also enable the identification of potential differences in eQTL effects between populations (GTEx Consortium, 2020) and detecting additional colocalization signals that can advance functional interpretation of GWAS loci (Mogil et al., 2018). Therefore, concerted efforts are warranted to increase the representation of diverse groups not only in GWAS, but also in tissue‐ and cell‐specific molecular databases to advance the interpretability of GWAS discoveries. Another area which can benefit significantly from data from diverse populations is fine‐mapping (Tehranchi et al., 2019). By applying different statistical methods, fine‐mapping utilizes LD structure and/or functional annotation to prioritize variants that are potentially causal which can be followed up in functional studies. Because diverse populations present divergent LD structures, multi‐ethnic fine‐mapping is superior in power to single‐ethnic analyses (Mahajan et al., 2022). There are several methods and tools available such as trans‐ethnic PAINTOR (Kichaev & Pasaniuc, 2015) and MsCAVIAR (LaPierre et al., 2021) that both use a multivariate normal distribution for modelling. MsCAVIAR additionally integrates functional annotation, MR‐MEGA applies trans‐ethnic mega regression (Magi et al., 2017).

To build more diverse and representative resources and databases, concerted efforts are needed. Methods adapted from the social science field can be used to explore recruitment strategies into genomics research for underrepresented research participants (Bull et al., 2012). A practical example in an African setting is an implementation of community‐level rapid assessment before a genomics research on podoconiosis (Tekola et al., 2009a, 2009b). The assessment revealed locally sensitive notions and concerns, which have been incorporated in recruitment and consent process strategies to minimize stigmatization of research participants, address cultural norms, which led to successful recruitment (Tekola Ayele et al., 2012). In their roadmap to increase diversity in genomic studies, Fatumo, Chikowore, et al. (2022) propose that building trust can promote the engagement of underrepresented participants in genomic research. Researchers can develop genuine partnerships with local communities and minority groups such as through community advisory boards that are sustained by community members. Funders can also play a pivotal role in promoting research in underrepresented populations. Strategic funding from the National Institutes of Health (NIH) and Wellcome Trust was successful in creating the H3Africa initiative. Strategic programs that can account for the needs of local research communities that struggle with lower competitive edges should be considered. The funding opportunities from research intensive countries should be more inclusive to applications from underrepresented populations and consider developing funding schemes that promote scientific networking and sustainability of the research capacity in those populations (Fatumo, Chikowore, et al., 2022).

## 5 BIOMEDICAL, CLINICAL, AND PUBLIC HEALTH PERSPECTIVES

The possibility of adapting PGS for clinical use is under consideration for some traits. However, the use of PGS in both research and clinical applications raise potential ethical concerns, such as the exacerbation of health inequities (see Section 3.5 for further discussion) (Martin et al., 2019; Palk et al., 2019; Polygenic Risk Score Task Force of the International Common Disease Alliance, 2021).

Scientific knowledge of the clinical significance of genetic variants, particularly rare variants, is not yet based on data from diverse populations. Most samples in the Genome Aggregation Database (gnomAD) (Karczewski et al., 2020) come from populations of European ancestry, and many of the variants listed as “pathogenic” or “likely pathogenic” in ClinVar (Popejoy et al., 2018) were identified in European ancestry populations. This has consequences for the translation to health care because clinically actionable genetic variants are often initially identified using in silico prediction tools that rely on population‐based datasets. When genomic data from a limited range of populations are used to develop in silico prediction pipelines, the accuracy of variant classification outside these populations may be poor, which can lead to incorrect molecular diagnostics. For example, genetic variants that were misclassified as pathogenic for hypertrophic cardiomyopathy and subsequently used in clinical decisions were later found to be common among African Americans, which led to a reversal of some clinical decisions (Manrai et al., 2016). The problem is of particular relevance for African populations because of the high genetic diversity of the African continent and for which rare pathogenic variants have not yet been included in databases. One example includes monogenic variants for hearing loss where a comparatively low number of variants have been identified for African compared to European populations (Wonkam & de Vries, 2020). Also, environment‐related selection gives rise to variants that are more relevant in specific groups such as African‐ancestry enriched variants related to sickle cell disease (Wonkam & de Vries, 2020), and *APOL1* variants that are thought to have undergone selection rising to high frequency in sub‐Saharan Africa to protect against a deadly form of sleeping sickness, but are associated with higher risk for chronic kidney disease (Genovese et al., 2010). Rare mutations that contribute to about half of congenital non‐syndromic hearing impairment in European populations are clustered in the *GJB2* gene, but these mutations have limited predictive relevance in sub‐Saharan African populations (Lebeko et al., 2016).

Pharmacogenomics has been successfully applied in clinical genetics. However, underrepresented populations have lower potential to benefit from predicted drug responses. In this regard, a recent GWAS on schizophrenia patients of African ancestry undergoing clozapine treatment showed a strong association of a regulatory variant in the *ACKR1* gene with the development of neutropenia, a serious adverse event that results in stopping the clozapine treatment (Bentley et al., 2020; M. H. Wang et al., 2019). This variant is rare in European or Asian ancestry individuals, and was only discovered because the GWAS was performed in African ancestry individuals.

Increasing the diversity of the biomedical research workforce can also contribute to improving scientific innovation derived from having different perspectives that promote creativity. Concerted efforts are being made to promote the training and support of scientists from underrepresented backgrounds working in the genomic field (Bonham & Green, 2021). Because groups who do not take part in genomic studies are less likely to benefit from medical advances based on genomics, it is imperative that the scientific community strives to be inclusive at all stages of study design, both in terms of study participants and researchers. Working to promote transparency, authors should describe clearly how diverse participants were included or explain why they were excluded from a study (Hindorff et al., 2018).

### 5.1 Limitations and interpretation issues

Interpretation of admixture mapping findings in the context of trait differences between populations warrants careful consideration of its assumptions and limitations. Ancestry estimation in admixed populations is error‐prone because the true historic ancestral parent populations are unknown and the availability of representative ancestral reference genetic data is limited for some populations (Baran et al., 2012). Therefore, replication in independent cohorts remains critical to validate associations identified by admixture mapping (Mersha, 2015). Regarding interpretation, it is important to bear in mind that allele frequency differences between geographically distant ancestral populations may be driven by confounding environmental factors unrelated to ancestry or the phenotype under consideration. An association of global ancestry with a phenotype may be partly or fully due to a confounding correlation of global ancestry with lifestyle and sociodemographic factors associated with the phenotype. Therefore, it is important to avoid generalizations that invoke “genetic” explanations for group differences based on observed associations of global ancestry with trait differences between population groups.

Genetic differences between individuals within the same population are often higher than differences between individuals from different populations (Rosenberg, 2011; Witherspoon et al., 2007). In addition, population differences are sometimes artificially enhanced by study design or analysis methods, for instance when studies investigating differences between populations exclude admixed participants, leading to exaggeration of population differences. Software designed to genetically differentiate populations may depend on the use of discrete populations, ignoring the possibility of clines (Lawson et al., 2018). Furthermore, the popular practice of removing “outliers,” for example, based on a PCA plot, can generate artificial population groupings and lead to the mistaken impression that populations are more genetically distinct than they actually are. Genetic population data should be reviewed to decide whether clusters or continuous clines adequately describe the population structure, taking external population information into account. This not only has implications for the statistical methods applied but also for the interpretation and communication of results. Indeed, scientists are encouraged to use precise terminology for population‐specific reference samples and avoid broad generalizations (Hunt & Megyesi, 2008).

## 6 CONCLUSIONS

The field of genetic epidemiology is undergoing a transformation with the collection of data with more diverse ancestral and social origins and with research staff and participants that are gradually becoming more inclusive. This shift is fuelled in part by concerted efforts toward the collection of diverse data at the study design level and as part of large reference data sets. We must be prepared to continue to support these efforts by engaging financially and scientifically in the years ahead to achieve broad data collections that will have meaningful impacts. The benefits of this shift toward more diversified genetic research include the potential for providing better treatment options to currently underrepresented communities, more equitable power dynamics with respect to health decisions and the potential for novel genetic discoveries that will be valuable to all communities, not just those where the discoveries are made.

To fully benefit from the growing admixed and diversified data collections, methodological tools should follow suit. We need to move beyond the traditional stratified datasets based on arbitrary population boundaries. We should support existing and future data analysts with documentation, training, and guidelines. As new models and approaches are being developed, our community should proceed with transparency following our hard‐earned standards of methodological rigor. Concerted efforts to evaluate and compare the performance and limitations of the various methods being developed will help to document, improve and set standards for the conduct of analyses with diverse and admixed genetic data sets.

## Data Availability

Data sharing not applicable to this article as no data sets were generated or analysed during the current study.
